# Disorder of the dimeric TCNQ–TCNQ unit in the crystal structure of [Ni(bpy)_3_]_2_(TCNQ–TCNQ)(TCNQ)_2_·6H_2_O (TCNQ is 7,7,8,8-tetra­cyano­quinodi­methane)

**DOI:** 10.1107/S2056989016019162

**Published:** 2017-01-01

**Authors:** Juraj Černák, Juraj Kuchár, Michal Hegedüs

**Affiliations:** a Department of Inorganic Chemistry, Institute of Chemistry, P. J. Šafárik University in Košice, Moyzesova 11, 041 54 Košice, Slovakia

**Keywords:** crystal structure, nickel, 2,2′-bi­pyridine, σ-dimerized TCNQ–TCNQ dianion, disorder

## Abstract

Crystallization from an aqueous methanol system composed of Ni(NO_3_)_2_, 2,2′-bipyridine (bpy) and LiTCNQ (TCNQ is 7,7,8,8-tetra­cyano­quinodi­methane) in a 1:3:2 molar ratio yielded single crystals of bis­[tris­(2,2′-bi­pyridine-κ^2^ *N*,*N*′)nickel(II)] bis­(7,7,8,8-tetra­cyano­quinodi­methane radical anion) bi[7,7,8,8-tetra­cyano­quino­dimethanide] hexa­hydrate, [Ni(C_10_H_8_N_2_)_3_]_2_(C_24_H_8_N_8_)(C_12_H_4_N_4_)_2_·6H_2_O or [Ni(bpy)_3_]_2_(TCNQ–TCNQ)(TCNQ)_2_·6H_2_O. The crystal structure comprises [Ni(bpy)_3_]^2+^ complex cations, two centrosymmetric crystallographically independent TCNQ^**·**−^ anion radicals with π-stacked exo groups, and an additional dimeric TCNQ–TCNQ unit which comprises 75.3 (9)% of a σ-dimerized (TCNQ–TCNQ)^2−^ dianion and 24.7 (9)% of two TCNQ^·−^ anion radicals with tightly π-stacked exo groups. The title complex represents the first example of an Ni^II^ complex containing a σ-dimerized (TCNQ–TCNQ)^2−^ dianion. Disordered solvent water mol­ecules present in the crystal structure participate in hydrogen-bonding inter­actions.

## Abstract 2

The first example of an Ni^II^ complex containing an σ-dimerized TCNQ–TCNQ unit is presented, with a C—C bond length of 1.653 (11) Å. In addition, the σ-dimerized TCNQ–TCNQ unit (refined 75% occupancy) is disordered, forming also a less populated pair of TCNQ mol­ecules (25% occupancy) with tightly π-stacked di­cyano­methanide groups.

## Chemical context   

In the quest for new promising mol­ecular magnetic materials besides the complexes of 3*d* and 4*f* elements, organic radicals have been explored (Nafady *et al.*, 2014 ▸; Kubota *et al.*, 2014 ▸; Starodub & Starodub, 2014 ▸). Among these, 7,7,8,8-tetra­cyano­quinodi­methane (TCNQ) in its anion radical form responds to magnetic probing. Its combination with 3*d* or 4*f* metal atoms may lead to inter­esting magnetic properties (Nishijo & Enomoto, 2015 ▸; Madalan *et al.*, 2002 ▸; Ballester *et al.*, 2002 ▸). In addition, materials containing TCNQ have been studied for their electric conductivity (Ballesteros-Rivas *et al.*, 2011 ▸; Starodub & Starodub, 2014 ▸). TCNQ (including its reduced forms), when combined with 3*d* metals, can be present as an non-coordinating species (in the neutral or anion radical form) or it can form a σ-bond with the metal atom (Ballester *et al.*, 1999 ▸). We note that TCNQ^.−^ anion radicals tend to dimerize, usually *via* stacking of their π-clouds, but, in some cases, the dimerization tendency leads to the formation of σ-dimerized (TCNQ–TCNQ)^2−^ dianions (Dong *et al.*, 1977 ▸; Hoffmann *et al.*, 1983 ▸; Shimomura *et al.*, 2010 ▸; Zhao *et al.*, 1996 ▸). Within our search for new heterospin materials based on 3*d* metals and organic radicals, we have undertaken a study of the aqueous methanol system containing Ni^II^, 2,2′-bi­pyridine (bpy) and TCNQ. Several complexes of Ni^II^-containing TCNQ species have been reported previously, *e.g.* [Ni(terpy)_2_](TCNQ)_2_ (terpy is 2,2′:6′,2′′-terpyridine) with non-coordinating π-dimerized anion radicals (Alonso *et al.*, 2005 ▸) or [Ni(cyclam)(TCNQ)_2_] (cyclam is 1,4,8,11-tetra­aza­cyclo­tetra­deca­ne) with σ-coordinating anion radicals (Ballester *et al.*, 1997 ▸). From a similar system with bpy, the formation of [Ni(bpy)_3_](TCNQ)_4_·(CH_3_)_2_CO was reported, along with the results of its crystal structure analysis (Vasylets *et al.*, 2014 ▸). Following our synthetic procedure, we have isolated single crystals of novel composition, *i.e.* [Ni(bpy)_3_]_2_(TCNQ–TCNQ)(TCNQ)_2_·6H_2_O (**1**) and report here its crystal structure.
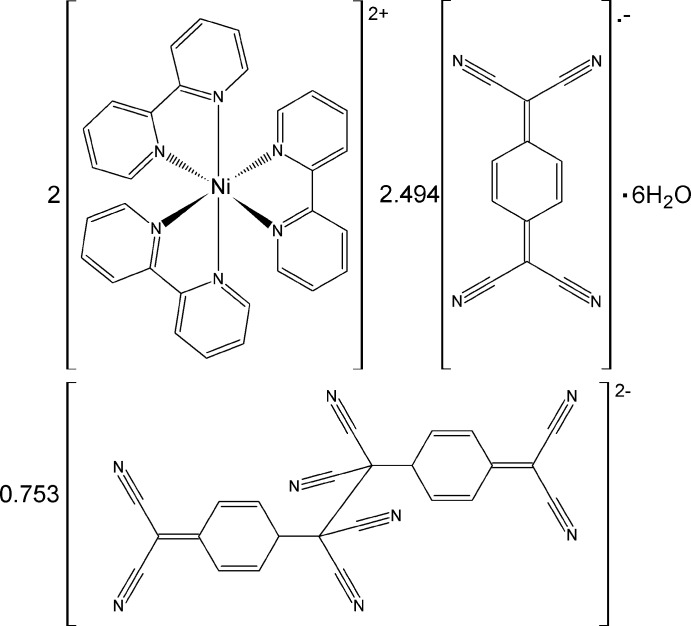


## Structural commentary   

**Figure 1:**
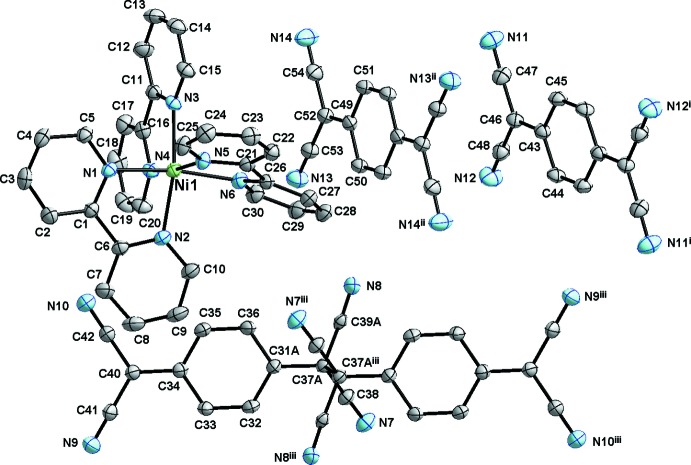
A view of the mol­ecular components of the title compound, **1**, showing the labelling and with displacement ellipsoids drawn at the 30% probability level. For the dimerized (TCNQ)_2_ unit, only the more populated position is shown. [Symmetry codes: (i) 1 − *x*, 2 − *y*, 1 − *z*; (ii) 1 − *x*, 1 − *y*, 1 − *z*; (iii) 1 − *x*, 1 − *y*, 2 − *z*.]

**Figure 2:**
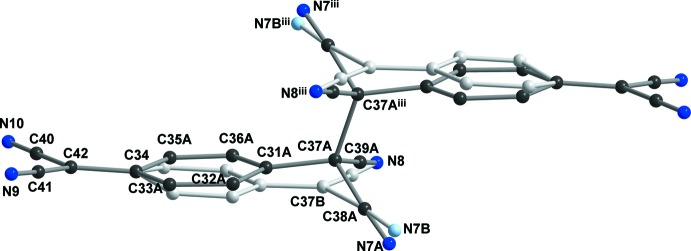
A view of the observed disorder of the dimerized (TCNQ)_2_ unit. The less populated atoms are shown with transparency. [Symmetry code: (iii) 1 − *x*, 1 − *y*, 2 − *z*.]

The unit cell of the title complex, **1**, comprises two [Ni(bpy)_3_]^2+^ complex cations, a centrosymmetric TCNQ–TCNQ dimeric unit, two centrosymmetric crystallographically independent TCNQ^·−^ anion radicals, and three crystallographically independent disordered solvent water mol­ecules (Figs. 1 ▸–5 ▸ ▸ ▸ ▸). The complex cation is optically active, but due to the centrosymmetric character of the space group, both Δ and Λ enanti­omers are present in the structure. The Ni—N bond lengths range from 2.078 (2) to 2.109 (2) Å. Similar values of 2.0895 (2) and 2.1023 (2) Å for Ni—N bonds were found in [Ni(bpy)_3_]_2_[W(CN)_8_]·6H_2_O (Korzeniak *et al.*, 2008 ▸). An outstanding feature of the structure of **1** is the presence of a σ-dimerized dianion TCNQA (Figs. 2 ▸ and 3 ▸), which is, to our knowledge, the first reported case of such a unit among Ni^II^ complexes with TCNQ. This dianion is disordered with a less prevalent pair of anion radicals for which the exocyclic groups inter­act solely *via* tight π-stacking, but are not σ-bonded; the refined site-occupation factors are 0.753 (9):0.247 (9) (Fig. 2 ▸). The simultaneous presence of both a σ-dimerized dianion and a pair of anion radicals can be considered as a manifestation of a not completed dimerization reaction. The C37*A*—C37*A* ^iii^ [symmetry code: (iii) 1 − *x*, 1 − *y*, 2 − *z*] dimerization bond length is 1.653 (11) Å and this value is within the usual range (see *Database survey* section). At the same time, this value is longer than a usual single C—C bond and, consequently, the corresponding bond angles around the C37*A* atom range from 105.6 (4) to 113.6 (3)°, displaying significant deviations from the ideal tetra­hedral angle. In the less populated pair of anion radicals within TCNQA, the distance between the C37*B* atom and its symmetry-related counterpart C37*B* ^iii^ is 3.06 (2) Å; the inter­planar distance between the least-squares plane P1 formed by atoms C31*B*, C37*B*, C38*B* and C39*B* and the least-squares plane P2 formed by their symmetry-related counterparts through a centre of symmetry at (1 − *x*, 1 − *y*, 2 − *z*) is 3.03 Å. The distance of the C37^iii^ atom from the plane P1 is 2.90 Å and the slippage between atoms C37*B* and C37*B* ^iii^ is 0.98 Å. These geometric parameters suggest a very strong π-inter­action between the less populated pair of anion radicals in TCNQA, and they are pre-positioned for σ-dimerization with little structural rearrangement required upon formation of the covalent bond. This could be seen as an indication of σ-bond formation in the solid state upon crystallization rather than pre-formation of the σ-dimers in solution.

**Figure 3:**
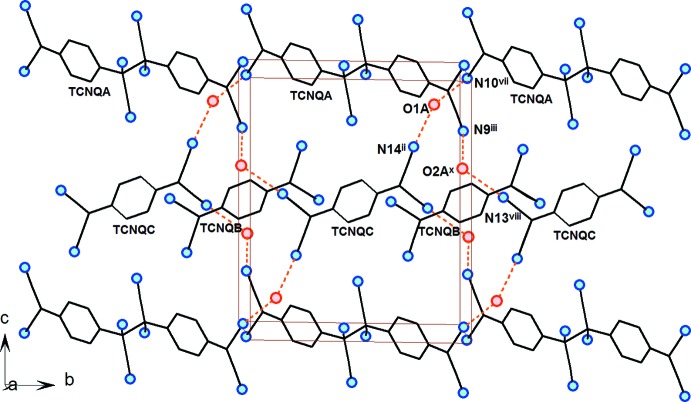
A view of the packing of the title compound, **1**, approximatively along the *a* axis. The complex cations, H atoms and O3 water mol­ecules have been omitted for clarity. Possible hydrogen bonds are shown as orange dashed lines. [Symmetry codes: (ii) 1 − *x*, 1 − *y*, 1 − *z*; (iii) 1 − *x*, 1 − *y*, 2 − *z*; (vii) *x*, 1 + *y*, *z*; (x) 1 + *x*, *y*, *z*.]

In addition to the TCNQA site, there are two crystallographically independent centrosymmetric TCNQ^·−^ anion radicals, TCNQB and TCNQC, in the crystal structure of **1** (Fig. 3 ▸). The two anion radicals are neighbours and stack in a π-stacked ‘external bond over external bond’ fashion (see Ballester *et al.*, 1999 ▸). The exocyclic groups in these TCNQ units are almost in plane with the quinoide ring; the greatest deviation from planarity is represented by the torsion angle C45—C43—C46—C48 of 175.9 (2)° in TCNQB.

## Supra­molecular features   

A view of the packing of the structure of **1** is displayed in Fig. 3 ▸. The TCNQ units are arranged in a chain-like manner along the *b* axis; one chain-like arrangement is formed only by the TCNQA dimeric units, while a second one is built up of alternating TCNQB and TCNQC anion radicals. In both chain-like arrangements, the exocyclic groups are π-stacked with each other. Ballester *et al.* (1999 ▸) defined four different stacking modes of TCNQ units, with typical intra­dimer distances between 3.09 and 3.45 Å. For TNCQA, the site with disordered σ-dimerized and radical anions, mol­ecules are arranged in infinite channels along a string of inversion centres on both sides of each crystallographically independent unit. On one side there is the case of the less populated un-σ-dimerized dianion, clearly a rather strong π-stacking inter­action (see above). The other side of the mol­ecule, involving the di­cyano­methanide group containing the C40 atom, on the other hand, stacks with its inversion-symmetry-related counterpart in an ‘external bond over external bond’ fashion defined as type ‘(*d*)’ by Ballester *et al.* (1999 ▸) (Fig. 3 ▸). The shortest observed distance of 3.54 (5) Å between atoms C33*B* ^iii^ and N10^vii^ [symmetry code: (vii) *x*, 1 + *y*, *z*] is, however, much longer than for the ‘front-end’ di­cyano­methanide group. It is outside the usually observed range for strong π-stacking inter­actions in analogous systems (Ballester *et al.*, 1999 ▸).

The mutual positions of the TCNQB and TCNQC anion radicals within the supra­molecular chain-like arrangement can be described as π-stacked in an ‘external bond over external bond’ fashion (Fig. 3 ▸), but we have to note that the TCNQB and TCNQC quinoide rings are not coplanar, as the least-squares planes through these quinoide rings form an angle of 9.42 (8)°. The shortest distance between the TCNQB and TCNQC anion radicals within the chain-like arrangement is 3.397 (4) Å [C46⋯C52^ii^; symmetry code: (ii) 1 − *x*, 1 − *y*, 1 − *z*] and the second shortest contact is 3.479 (4) Å between atoms C46 and C53^ii^; the latter distance is already somewhat longer due to the noncoplanarity of the two anion radicals. These observed distances are at the upper border for stacking arrangements reported for similar compounds (Ballester *et al.*, 1999 ▸).

**Figure 5:**
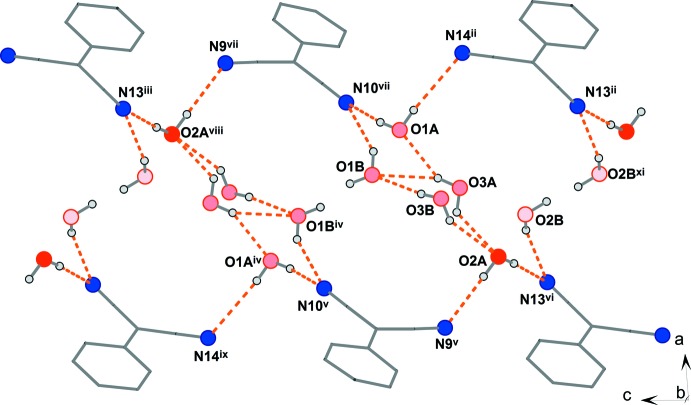
A view of the possible hydrogen-bonding system in the crystal structure of the title complex, **1**. Hydrogen bonds are represented by orange dashed lines. The different undertones of the red colour used for the O atoms reflect the value of the site-occupation factor (sof): dark-red (O2*A*): sof = 0.908 (3); light-red (O2*B*): 0.092 (3); inter­mediate (O1*A* and O2*A*): exactly 0.5. [Symmetry codes: (ii) 1 − *x*, 1 − *y*, 1 − *z*; (iii) 1 − *x*, 1 − *y*, 2 − *z*; (iv) −*x*, 2 − *y*, 2 − *z*; (v) −*x*, 1 − *y*, 2 − *z*; (vi) *x* − 1, 1 + *y*, *z*; (vii) *x*, 1 + *y*, *z*; (viii) −*x*, 2 − *y*, 2 − *z*; (ix) *x* − 1, 1 + *y*, 1 + *z*; (xi) −*x*, 2 − *y*, 1 − *z*.]

There are three crystallographically independent positionally disordered water solvent mol­ecules in the structure which, through the formation of O—H⋯O and O—H⋯N hydrogen bonds, play an important role in the formation of the supra­molecular structure of **1** (Figs. 3 ▸, 4 ▸ and 5 ▸, and Table 1 ▸). Water mol­ecules O1*A* and O2*A* are linked *via* N⋯H—O—H⋯N (the N atoms are from the nitrile groups of the TCNQ units) hydrogen-bonded bridges involving TCNQA dianions and TCNQC anion radicals, yielding a supra­molecular layer within the *bc* plane (Figs. 3 ▸ and 4 ▸). In addition, these supra­molecular layers are inter­connected by O2*A*⋯H—O3*A*—H⋯O1*A* hydrogen-bonded bridges, resulting in a three-dimensional hydrogen-bonded supra­molecular structure. We note that atoms O1*A*, O2*A* and O3*A* are only partially occupied due to the observed disorder. The alternatively positioned O1 and O3 water mol­ecules (disordered positions O1*B* and O3*B*) form an additional hydrogen-bonded bridging path, N⋯H—O2*A*⋯H—O3*B*—H⋯O1*B*—H⋯N, between the supra­molecular layers. On the other hand, the least-occupied position (O2*B*) of water mol­ecule O2 seems to be hydrogen bonded only to the nitrile N atom and so partially occupies the void in the structure in alternation with its symmetry-related atom O2*B* ^xi^ [symmetry code: (xi) −*x*, 2 − *y*, 1 − *z*] (Fig. 5 ▸). Additional weak hydrogen-bonding inter­actions of the C—H⋯N and C—H⋯O types (Table 1 ▸) contribute to the stability of the structure.

## Database survey   

A search of the CSD (Groom *et al.*, 2016 ▸) revealed 16 compounds with σ-dimerized TCNQ–TCNQ units. Among the hits in the CSD with σ-dimerized TCNQ–TCNQ dianions, there is no example containing an Ni^II^ ion as the central atom, hence compound **1** is the first such example. The reported values of the C—C bond linking the two TCNQ units are slightly longer than a normal single bond; the reported values range from 1.612 Å, found in *catena*-[Zn(TCNQ–TCNQ)(bipy)]·p-xy (bipy is 4,4′-bi­pyridine and p-xy is *p*-xylene; Shimomura *et al.*, 2010 ▸), to 1.673 Å, found in [Pt(bpy)_2_)(TCNQ–TCNQ)] (Dong *et al.*, 1977 ▸). In **1**, the corresponding value is 1.653 (11) Å, which is in line with the observed range in the published crystal structures.

## Synthesis and crystallization   

A solution of LiTCNQ (0.150 mmol, 31.6 mg) in methanol (2 ml) heated to 323 K was added dropwise to a mixture of Ni(NO_3_)_2_·6H_2_O (0.075 mmol, 21.8 mg) and bpy (0.225 mmol, 35.1 mg) in methanol (2 ml) at the same temperature. The dark-green solution that resulted was immediately enclosed in a 5 ml vial and cooled to room temperature (8.75 K h^−1^) in a programmable drying oven. The dark-green crystalline solid that resulted was filtered off, washed with a small amount of methanol and ether, and dried in air. The solid was mainly of microcrystalline character, with a few single crystals suitable for X-ray study (yield 63%). IR (PerkinElmer Spectrum 100 FT–IR Spectrophotometer with a UATR accessory in the range 4000–400 cm^−1^, KBr, cm^−1^): 3341 (*m*), 3382 (*m*), 3074 (*vw*), 3033 (*vw*), 2200 (*s*), 2175 (*vs*), 2152ssh, 1598 (*m*), 1581 (*s*), 1504 (*s*), 1471 (*m*), 1441 (*m*), 1359 (*s*), 1182 (*m*), 1020 (*w*), 987 (*w*), 826 (*w*), 779 (*m*), 765 (*m*), 737 (*w*), 653 (*w*), 483 (*w*). CNH (CHNOS Elemental Analyzer vario MICRO instrument; calculated/experimental, %): C 65.54/67.00, H 3.87/3.98, N 19.81/19.80.

## Refinement   

Crystal data, data collection and structure refinement details are summarized in Table 2 ▸. H atoms bound to C atoms were positioned in calculated positions, with their *U*_iso_ values set at 1.2 times the *U*_eq_ value of the parent C atom. During refinement it became apparent that what initially was considered as only a σ-dimerized (TCNQ–TCNQ)^2−^ dianion is positionally disordered (see Fig. 2 ▸); it consists mostly of a σ-dimerized dianion disordered with a less abundant dimeric unit having closly π-stacked di­cyano­methanide groups. The effort to resolve this disorder yielded refined site-occupation factors of 0.753 (9):0.247 (9). The observed disorder involves the di­cyano­methanide group involved in dimerization, as well as the quinoide ring atoms with the exception of atom C34. In order to control the geometric parameters, the disordered quinoide ring atoms, as well as the C37 atoms of each disordered moiety, were restrained to be coplanar (FLAT command) and equivalent bond lengths of disordered atoms were restrained to be similar (SADI commands). The refinement process concerning the solvent water mol­ecules was carried out using an iterative approach which showed that there are three crystallographically independent water mol­ecules in the asymmetric unit and that all of them are positionally disordered; some of the disorder is symmetry imposed, with atoms related through a centre of symmetry being mutually exclusive due to close contacts, and the site-occupation factors for these atoms (O1*A*, O1*B*, O3*A* and O3*B*) were considered to be exactly one half, while the refined site-occupation factors for atoms O2*A* and O2*B* are 0.908 (3) and 0.092 (3), respectively. Some of the water H atoms were resolved in difference maps and all H-atom positions were refined assuming idealized geometric parameters of O—H = 0.85 (1) Å and H⋯H = 1.344 (1) Å. For the H atoms of the O2*B* water mol­ecule (the least-occupied water mol­ecule), a riding model was used. The *U*_iso_ parameters for water H atoms were set at 1.5 times the *U*_eq_ value of the parent O atom.

## Supplementary Material

Crystal structure: contains datablock(s) I. DOI: 10.1107/S2056989016019162/zl2686sup1.cif

Structure factors: contains datablock(s) I. DOI: 10.1107/S2056989016019162/zl2686Isup2.hkl

CCDC reference: 1520298

Additional supporting information: crystallographic information; 3D view; checkCIF report

## Figures and Tables

**Figure 4:**
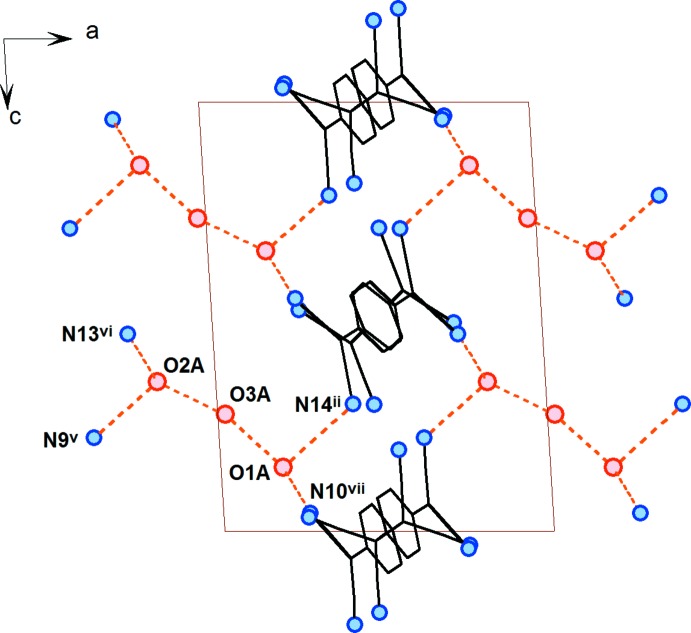
A view of the packing along the *b* axis, showing the role of the O3*A* water mol­ecule in linking the supra­molecular sheets into a three-dimensional supra­molecular network. The complex cations and H atoms have been omitted for clarity. Possible hydrogen bonds are shown as orange dashed lines. [Symmetry codes: (ii) 1 − *x*, 1 − *y*, 1 − *z*; (v) −*x*, 1 − *y*, 2 − *z*; (vi) *x* − 1, 1 + *y*, *z*; (vii) *x*, 1 + *y*, *z*; (x) 1 + *x*, *y*, *z*.]

**Table 1:** Hydrogen-bond geometry (Å, °)

*D*—H⋯*A*	*D*—H	H⋯*A*	*D*⋯*A*	*D*—H⋯*A*
O1*A*—H1*A*⋯N14^i^	0.84 (1)	2.60 (2)	3.438 (8)	174 (8)
O1*A*—H1*B*⋯N10^ii^	0.84 (1)	2.14 (2)	2.933 (5)	159 (5)
O1*B*—H1*C*⋯N10^ii^	0.84 (1)	2.11 (2)	2.865 (5)	150 (4)
O2*A*—H2*A*⋯N9^iii^	0.85 (1)	2.22 (1)	3.068 (4)	178 (4)
O2*A*—H2*B*⋯N13^iv^	0.85 (1)	2.15 (1)	2.993 (4)	173 (4)
O2*B*—H2*C*⋯N13^iv^	0.85	2.05	2.803 (16)	147
O3*A*—H3*A*⋯O2*A*	0.85 (1)	2.09 (2)	2.71 (2)	130 (3)
O3*A*—H3*B*⋯O1*A*	0.85 (1)	2.09 (2)	2.85 (2)	147 (5)
O3*A*—H3*B*⋯O1*B*	0.85 (1)	2.39 (4)	3.20 (2)	160 (6)
O3*B*—H3*C*⋯O1*B*	0.85 (1)	1.99 (2)	2.84 (2)	170 (9)
O3*B*—H3*D*⋯O2*A*	0.85 (1)	2.10 (2)	2.864 (16)	148 (5)
C4—H4⋯N11^v^	0.95	2.58	3.350 (3)	138
C5—H5⋯N3	0.95	2.67	3.213 (3)	117
C7—H7⋯O1*B* ^iii^	0.95	2.53	3.418 (7)	156
C10—H10⋯N6	0.95	2.63	3.168 (3)	116
C12—H12⋯O2*B* ^v^	0.95	2.44	3.30 (2)	150
C15—H15⋯N5	0.95	2.65	3.188 (3)	117
C15—H15⋯N12^vi^	0.95	2.68	3.369 (4)	130
C20—H20⋯N2	0.95	2.69	3.227 (3)	116
C22—H22⋯O3*B*	0.95	2.48	3.366 (15)	155
C25—H25⋯N8^vi^	0.95	2.49	3.184 (3)	130
C27—H27⋯O3*A*	0.95	2.55	3.43 (2)	155
C27—H27⋯O3*B*	0.95	2.33	3.276 (18)	172
C29—H29⋯N8	0.95	2.67	3.432 (3)	137
C29—H29⋯N11^i^	0.95	2.69	3.295 (4)	123
C30—H30⋯N11^i^	0.95	2.63	3.279 (4)	126

Symmetry codes: (i) 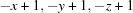; (ii) 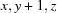; (iii) 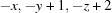; (iv) 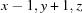; (v) 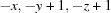; (vi) 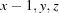.

**Table 2:** Experimental details

Crystal data	
Chemical formula	[Ni(C_10_H_8_N_2_)_3_]_2_(C_24_H_8_N_8_)(C_12_H_4_N_4_)_2_·6H_2_O
*M*_r_	1979.35
Crystal system, space group	Triclinic, *P* 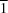
Temperature (K)	200
*a*, *b*, *c* (Å)	12.4034 (4), 13.2921 (4), 15.4869 (4)
α, β, γ (°)	88.828 (3), 86.336 (3), 73.586 (3)
*V* (Å^3^)	2444.21 (13)
*Z*	1
Radiation type	Mo *K*α
μ (mm^−1^)	0.46
Crystal size (mm)	0.52 × 0.39 × 0.28

Data collection	
Diffractometer	Rigaku OD Xcalibur, Sapphire2, large Be window
Absorption correction	Analytical [*CrysAlis PRO* (Rigaku OD, 2015 ▸), based on expressions derived by Clark & Reid (1995 ▸)]
*T*_min_, *T*_max_	0.864, 0.914
No. of measured, independent and observed [*I* > 2σ(*I*)] reflections	31246, 11255, 7475
*R*_int_	0.035
(sin θ/λ)_max_ (Å^−1^)	0.681

Refinement	
*R*[*F* ^2^ > 2σ(*F* ^2^)], *wR*(*F* ^2^), *S*	0.049, 0.126, 1.05
No. of reflections	11255
No. of parameters	726
No. of restraints	37
H-atom treatment	H atoms treated by a mixture of independent and constrained refinement
Δρ_max_, Δρ_min_ (e Å^−3^)	0.34, −0.24

Computer programs: *CrysAlis PRO* (Rigaku OD, 2015 ▸), *SIR92* (Altomare *et al.*, 1994 ▸), *SHELXL2014* (Sheldrick, 2015 ▸), *DIAMOND* (Brandenburg, 2007 ▸) and *publCIF* (Westrip, 2010 ▸).

## supplementary crystallographic information

### Crystal data

**Table:**

[Ni(C_10_H_8_N_2_)_3_]_2_(C_24_H_8_N_8_)(C_12_H_4_N_4_)_2_·6H_2_O	*Z* = 1
*M**_r_* = 1979.35	*F*(000) = 1024
Triclinic, *P*1	*D*_x_ = 1.345 Mg m^−^^3^
*a* = 12.4034 (4) Å	Mo *K*α radiation, λ = 0.71073 Å
*b* = 13.2921 (4) Å	Cell parameters from 10858 reflections
*c* = 15.4869 (4) Å	θ = 3.4–3.4°
α = 88.828 (3)°	µ = 0.46 mm^−^^1^
β = 86.336 (3)°	*T* = 200 K
γ = 73.586 (3)°	Prism, green
*V* = 2444.21 (13) Å^3^	0.52 × 0.39 × 0.28 mm

### Data collection

**Table:**

Rigaku OD Xcalibur, Sapphire2, large Be window diffractometer	11255 independent reflections
Radiation source: fine-focus sealed X-ray tube	7475 reflections with *I* > 2σ(*I*)
Detector resolution: 8.3438 pixels mm^-1^	*R*_int_ = 0.035
ω scans	θ_max_ = 29.0°, θ_min_ = 2.9°
Absorption correction: analytical [CrysAlis PRO (Rigaku OD, 2015), based on expressions derived by Clark & Reid (1995)]	*h* = −16→16
*T*_min_ = 0.864, *T*_max_ = 0.914	*k* = −18→17
31246 measured reflections	*l* = −20→19

### Refinement

**Table:**

Refinement on *F*^2^	37 restraints
Least-squares matrix: full	Hydrogen site location: mixed
*R*[*F*^2^ > 2σ(*F*^2^)] = 0.049	H atoms treated by a mixture of independent and constrained refinement
*wR*(*F*^2^) = 0.126	*w* = 1/[σ^2^(*F*_o_^2^) + (0.050*P*)^2^ + 0.5977*P*] where *P* = (*F*_o_^2^ + 2*F*_c_^2^)/3
*S* = 1.05	(Δ/σ)_max_ < 0.001
11255 reflections	Δρ_max_ = 0.34 e Å^−^^3^
726 parameters	Δρ_min_ = −0.24 e Å^−^^3^

### Special details

**Table:**

Geometry. All esds (except the esd in the dihedral angle between two l.s. planes) are estimated using the full covariance matrix. The cell esds are taken into account individually in the estimation of esds in distances, angles and torsion angles; correlations between esds in cell parameters are only used when they are defined by crystal symmetry. An approximate (isotropic) treatment of cell esds is used for estimating esds involving l.s. planes.

### Fractional atomic coordinates and isotropic or equivalent isotropic displacement parameters (Å^2^)

**Table:**

	*x*	*y*	*z*	*U*_iso_*/*U*_eq_	Occ. (<1)
O1A	0.1896 (5)	0.8735 (5)	0.8522 (4)	0.1007 (18)	0.5
H1A	0.247 (4)	0.847 (8)	0.820 (5)	0.151*	0.5
H1B	0.210 (7)	0.903 (5)	0.893 (2)	0.151*	0.5
O1B	0.0587 (5)	0.9770 (6)	0.9161 (4)	0.129 (2)	0.5
H1C	0.125 (4)	0.982 (9)	0.909 (3)	0.193*	0.5
H1D	0.033 (6)	1.005 (11)	0.964 (5)	0.193*	0.5
O2A	−0.1755 (2)	0.9694 (2)	0.65321 (18)	0.0834 (8)	0.908 (3)
H2A	−0.232 (2)	0.975 (3)	0.689 (2)	0.125*	0.908 (3)
H2B	−0.197 (3)	1.020 (2)	0.618 (2)	0.125*	0.908 (3)
O2B	−0.0574 (17)	1.0092 (17)	0.5847 (16)	0.0834 (8)	0.092 (3)
H2C	−0.1045	1.0697	0.5835	0.125*	0.092 (3)
H2D	−0.0210	1.0037	0.5357	0.125*	0.092 (3)
O3A	0.0233 (18)	0.8983 (17)	0.7290 (12)	0.186 (7)	0.5
H3A	−0.047 (2)	0.924 (13)	0.739 (2)	0.279*	0.5
H3B	0.051 (5)	0.911 (10)	0.775 (5)	0.279*	0.5
O3B	−0.0063 (16)	0.8831 (12)	0.7729 (13)	0.155 (7)	0.5
H3C	0.007 (9)	0.918 (8)	0.814 (7)	0.232*	0.5
H3D	−0.069 (5)	0.920 (10)	0.756 (3)	0.232*	0.5
Ni1	0.00252 (2)	0.36352 (2)	0.74798 (2)	0.03705 (10)	
N1	−0.11257 (15)	0.28116 (15)	0.78825 (12)	0.0382 (4)	
N2	0.02588 (16)	0.34809 (16)	0.88183 (12)	0.0415 (5)	
N3	−0.01954 (16)	0.34866 (16)	0.61637 (12)	0.0399 (5)	
N4	0.12404 (16)	0.22081 (16)	0.71749 (12)	0.0411 (5)	
N5	−0.11165 (15)	0.51236 (16)	0.75713 (12)	0.0403 (5)	
N6	0.10952 (16)	0.45865 (16)	0.73098 (12)	0.0418 (5)	
C1	−0.11042 (19)	0.25284 (19)	0.87224 (15)	0.0392 (5)	
C2	−0.1837 (2)	0.2005 (2)	0.90954 (17)	0.0535 (7)	
H2	−0.1810	0.1814	0.9690	0.064*	
C3	−0.2613 (2)	0.1765 (2)	0.85878 (19)	0.0582 (7)	
H3	−0.3130	0.1416	0.8834	0.070*	
C4	−0.2627 (2)	0.2036 (2)	0.77311 (18)	0.0519 (7)	
H4	−0.3146	0.1872	0.7371	0.062*	
C5	−0.1865 (2)	0.25578 (19)	0.74003 (17)	0.0445 (6)	
H5	−0.1870	0.2742	0.6804	0.053*	
C6	−0.0262 (2)	0.28306 (19)	0.92217 (15)	0.0403 (5)	
C7	−0.0015 (2)	0.2457 (2)	1.00526 (16)	0.0583 (7)	
H7	−0.0385	0.1988	1.0325	0.070*	
C8	0.0777 (3)	0.2778 (3)	1.04744 (18)	0.0666 (9)	
H8	0.0979	0.2513	1.1033	0.080*	
C9	0.1271 (2)	0.3487 (3)	1.00788 (18)	0.0620 (8)	
H9	0.1791	0.3743	1.0369	0.074*	
C10	0.0994 (2)	0.3815 (2)	0.92542 (17)	0.0530 (7)	
H10	0.1339	0.4301	0.8979	0.064*	
C11	0.0359 (2)	0.25522 (19)	0.58218 (15)	0.0404 (5)	
C12	0.0153 (3)	0.2252 (2)	0.50062 (17)	0.0587 (7)	
H12	0.0555	0.1585	0.4776	0.070*	
C13	−0.0648 (3)	0.2945 (3)	0.45379 (18)	0.0664 (9)	
H13	−0.0827	0.2747	0.3991	0.080*	
C14	−0.1182 (2)	0.3921 (3)	0.48679 (17)	0.0602 (8)	
H14	−0.1711	0.4419	0.4543	0.072*	
C15	−0.0937 (2)	0.4167 (2)	0.56792 (16)	0.0490 (6)	
H15	−0.1306	0.4844	0.5906	0.059*	
C16	0.1199 (2)	0.18548 (19)	0.63741 (16)	0.0414 (6)	
C17	0.1909 (2)	0.0903 (2)	0.60849 (19)	0.0564 (7)	
H17	0.1860	0.0656	0.5521	0.068*	
C18	0.2686 (3)	0.0322 (2)	0.6630 (2)	0.0654 (8)	
H18	0.3192	−0.0325	0.6438	0.078*	
C19	0.2733 (2)	0.0672 (2)	0.7442 (2)	0.0627 (8)	
H19	0.3263	0.0275	0.7824	0.075*	
C20	0.1987 (2)	0.1624 (2)	0.76974 (18)	0.0530 (7)	
H20	0.2008	0.1868	0.8266	0.064*	
C21	−0.0658 (2)	0.59287 (19)	0.74590 (14)	0.0389 (5)	
C22	−0.1307 (2)	0.6948 (2)	0.73577 (16)	0.0463 (6)	
H22	−0.0963	0.7499	0.7271	0.056*	
C23	−0.2465 (2)	0.7157 (2)	0.73844 (17)	0.0527 (7)	
H23	−0.2927	0.7853	0.7311	0.063*	
C24	−0.2941 (2)	0.6348 (2)	0.75180 (17)	0.0512 (7)	
H24	−0.3735	0.6476	0.7552	0.061*	
C25	−0.2239 (2)	0.5343 (2)	0.76024 (17)	0.0480 (6)	
H25	−0.2569	0.4782	0.7686	0.058*	
C26	0.0594 (2)	0.5614 (2)	0.74387 (14)	0.0399 (5)	
C27	0.1211 (2)	0.6318 (2)	0.75518 (16)	0.0496 (6)	
H27	0.0845	0.7038	0.7662	0.059*	
C28	0.2382 (2)	0.5942 (3)	0.75003 (17)	0.0577 (8)	
H28	0.2827	0.6406	0.7582	0.069*	
C29	0.2888 (2)	0.4909 (3)	0.73328 (17)	0.0566 (7)	
H29	0.3685	0.4648	0.7274	0.068*	
C30	0.2221 (2)	0.4246 (2)	0.72501 (16)	0.0510 (7)	
H30	0.2574	0.3522	0.7147	0.061*	
N8	0.54409 (19)	0.48344 (18)	0.81067 (14)	0.0533 (6)	
N9	0.3772 (3)	0.0161 (2)	1.21738 (16)	0.0813 (9)	
N10	0.2633 (2)	0.0190 (2)	0.95837 (16)	0.0712 (8)	
C34	0.42800 (19)	0.16964 (19)	1.03807 (14)	0.0385 (5)	
N7A	0.7420 (5)	0.4375 (8)	1.0324 (8)	0.0541 (15)	0.753 (9)
C31A	0.5096 (4)	0.3468 (4)	0.9968 (3)	0.0344 (10)	0.753 (9)
C32A	0.5305 (7)	0.2926 (7)	1.0769 (5)	0.0389 (13)	0.753 (9)
H32A	0.5740	0.3147	1.1170	0.047*	0.753 (9)
C33A	0.4886 (9)	0.2079 (16)	1.0975 (10)	0.0393 (18)	0.753 (9)
H33A	0.5009	0.1752	1.1526	0.047*	0.753 (9)
C35A	0.4121 (11)	0.2223 (7)	0.9579 (4)	0.0415 (17)	0.753 (9)
H35A	0.3727	0.1977	0.9161	0.050*	0.753 (9)
C36A	0.4508 (6)	0.3076 (5)	0.9373 (5)	0.0389 (13)	0.753 (9)
H36A	0.4377	0.3402	0.8823	0.047*	0.753 (9)
C37A	0.5439 (3)	0.4471 (4)	0.9774 (2)	0.0368 (10)	0.753 (9)
C38A	0.6576 (2)	0.4385 (2)	1.00707 (16)	0.0434 (6)	0.753 (9)
C39A	0.5463 (7)	0.4688 (4)	0.8835 (3)	0.0334 (12)	0.753 (9)
N7B	0.725 (2)	0.460 (3)	1.041 (3)	0.0541 (15)	0.247 (9)
C31B	0.5328 (15)	0.3184 (15)	0.9834 (12)	0.0344 (10)	0.247 (9)
C32B	0.549 (3)	0.277 (3)	1.0618 (17)	0.0389 (13)	0.247 (9)
H32B	0.5941	0.3010	1.0993	0.047*	0.247 (9)
C33B	0.504 (3)	0.202 (5)	1.088 (3)	0.0393 (18)	0.247 (9)
H33B	0.5227	0.1687	1.1422	0.047*	0.247 (9)
C35B	0.423 (4)	0.203 (3)	0.9511 (13)	0.0415 (17)	0.247 (9)
H35B	0.3886	0.1719	0.9105	0.050*	0.247 (9)
C36B	0.470 (2)	0.2819 (19)	0.926 (2)	0.0389 (13)	0.247 (9)
H36B	0.4596	0.3116	0.8702	0.047*	0.247 (9)
C37B	0.5775 (11)	0.4002 (12)	0.9580 (8)	0.0368 (10)	0.247 (9)
C38B	0.6576 (2)	0.4385 (2)	1.00707 (16)	0.0434 (6)	0.247 (9)
C39B	0.561 (3)	0.4429 (15)	0.8746 (11)	0.0334 (12)	0.247 (9)
C40	0.3769 (2)	0.08793 (19)	1.06320 (15)	0.0437 (6)	
C41	0.3786 (3)	0.0475 (2)	1.14731 (17)	0.0529 (7)	
C42	0.3148 (2)	0.0497 (2)	1.00509 (16)	0.0481 (6)	
N11	0.5248 (2)	0.7399 (2)	0.29382 (17)	0.0701 (7)	
N12	0.7339 (2)	0.6518 (2)	0.51503 (18)	0.0747 (8)	
C43	0.5416 (2)	0.8954 (2)	0.46974 (15)	0.0431 (6)	
C44	0.5744 (2)	0.9267 (2)	0.54848 (16)	0.0477 (6)	
H44	0.6255	0.8763	0.5817	0.057*	
C45	0.4651 (2)	0.9737 (2)	0.42244 (16)	0.0475 (6)	
H45	0.4412	0.9556	0.3693	0.057*	
C46	0.5846 (2)	0.7918 (2)	0.43755 (17)	0.0479 (6)	
C47	0.5512 (2)	0.7624 (2)	0.35862 (19)	0.0508 (6)	
N13	0.7442 (2)	0.1592 (2)	0.54178 (17)	0.0708 (7)	
N14	0.5887 (3)	0.2293 (2)	0.29481 (19)	0.0916 (10)	
C48	0.6669 (2)	0.7136 (2)	0.48084 (18)	0.0531 (7)	
C49	0.5579 (2)	0.3964 (2)	0.47270 (16)	0.0490 (7)	
C50	0.5688 (2)	0.4322 (2)	0.55632 (16)	0.0523 (7)	
H50	0.6156	0.3859	0.5950	0.063*	
C51	0.4861 (2)	0.4685 (2)	0.41768 (16)	0.0518 (7)	
H51	0.4763	0.4468	0.3614	0.062*	
C52	0.6141 (2)	0.2929 (2)	0.44541 (17)	0.0539 (7)	
C53	0.6858 (2)	0.2194 (2)	0.49858 (18)	0.0549 (7)	
C54	0.6000 (3)	0.2571 (2)	0.3622 (2)	0.0646 (8)	

### Atomic displacement parameters (Å^2^)

**Table:**

	*U*^11^	*U*^22^	*U*^33^	*U*^12^	*U*^13^	*U*^23^
O1A	0.135 (5)	0.103 (4)	0.097 (4)	−0.076 (4)	−0.062 (3)	0.024 (3)
O1B	0.111 (5)	0.151 (6)	0.161 (6)	−0.089 (5)	−0.067 (4)	0.062 (5)
O2A	0.095 (2)	0.0602 (16)	0.086 (2)	−0.0038 (15)	−0.0259 (15)	0.0048 (14)
O2B	0.095 (2)	0.0602 (16)	0.086 (2)	−0.0038 (15)	−0.0259 (15)	0.0048 (14)
O3A	0.167 (11)	0.162 (14)	0.248 (18)	−0.060 (9)	−0.114 (12)	0.053 (10)
O3B	0.159 (12)	0.082 (5)	0.250 (17)	−0.054 (6)	−0.131 (12)	0.071 (8)
Ni1	0.03132 (16)	0.04012 (19)	0.03937 (17)	−0.00905 (13)	−0.00397 (12)	−0.00099 (13)
N1	0.0336 (10)	0.0374 (11)	0.0430 (11)	−0.0081 (9)	−0.0075 (8)	−0.0009 (9)
N2	0.0373 (11)	0.0474 (12)	0.0413 (11)	−0.0134 (10)	−0.0060 (9)	−0.0056 (9)
N3	0.0379 (11)	0.0399 (12)	0.0394 (11)	−0.0066 (9)	−0.0059 (9)	0.0054 (9)
N4	0.0360 (11)	0.0432 (12)	0.0420 (11)	−0.0071 (9)	−0.0057 (9)	0.0030 (9)
N5	0.0311 (10)	0.0421 (12)	0.0478 (11)	−0.0109 (9)	0.0004 (9)	−0.0019 (9)
N6	0.0325 (10)	0.0486 (13)	0.0442 (11)	−0.0111 (10)	−0.0015 (9)	−0.0020 (9)
C1	0.0398 (13)	0.0367 (13)	0.0412 (13)	−0.0106 (11)	−0.0025 (10)	−0.0031 (10)
C2	0.0614 (17)	0.0563 (17)	0.0477 (15)	−0.0254 (15)	−0.0009 (13)	0.0012 (13)
C3	0.0547 (17)	0.0537 (17)	0.074 (2)	−0.0296 (15)	0.0025 (15)	0.0002 (15)
C4	0.0458 (15)	0.0441 (15)	0.0702 (18)	−0.0172 (13)	−0.0140 (13)	−0.0056 (13)
C5	0.0413 (14)	0.0438 (15)	0.0502 (14)	−0.0127 (12)	−0.0132 (11)	0.0006 (12)
C6	0.0389 (13)	0.0442 (14)	0.0367 (12)	−0.0095 (11)	−0.0019 (10)	−0.0055 (11)
C7	0.0668 (19)	0.073 (2)	0.0397 (14)	−0.0265 (16)	−0.0074 (13)	0.0007 (14)
C8	0.073 (2)	0.090 (2)	0.0379 (15)	−0.0221 (19)	−0.0156 (14)	−0.0043 (15)
C9	0.0554 (17)	0.087 (2)	0.0486 (16)	−0.0246 (17)	−0.0145 (14)	−0.0170 (15)
C10	0.0467 (15)	0.0690 (19)	0.0499 (15)	−0.0250 (14)	−0.0078 (12)	−0.0104 (14)
C11	0.0396 (13)	0.0432 (14)	0.0385 (12)	−0.0127 (12)	−0.0004 (10)	0.0023 (11)
C12	0.0697 (19)	0.0617 (19)	0.0438 (15)	−0.0160 (16)	−0.0050 (14)	−0.0070 (13)
C13	0.082 (2)	0.081 (2)	0.0377 (15)	−0.0225 (19)	−0.0177 (15)	0.0044 (15)
C14	0.0613 (18)	0.073 (2)	0.0451 (15)	−0.0142 (16)	−0.0168 (14)	0.0167 (15)
C15	0.0464 (14)	0.0493 (16)	0.0472 (14)	−0.0067 (13)	−0.0068 (12)	0.0086 (12)
C16	0.0381 (13)	0.0383 (14)	0.0460 (14)	−0.0092 (11)	0.0013 (11)	0.0016 (11)
C17	0.0571 (17)	0.0441 (16)	0.0621 (17)	−0.0053 (14)	0.0000 (14)	−0.0055 (13)
C18	0.0598 (19)	0.0400 (16)	0.083 (2)	0.0053 (14)	0.0029 (16)	0.0020 (15)
C19	0.0479 (16)	0.0545 (18)	0.074 (2)	0.0051 (14)	−0.0104 (15)	0.0178 (16)
C20	0.0439 (15)	0.0562 (18)	0.0529 (16)	−0.0033 (13)	−0.0116 (12)	0.0077 (13)
C21	0.0419 (13)	0.0415 (14)	0.0347 (12)	−0.0136 (12)	−0.0043 (10)	−0.0030 (10)
C22	0.0517 (16)	0.0424 (15)	0.0461 (14)	−0.0146 (13)	−0.0067 (12)	−0.0015 (11)
C23	0.0541 (16)	0.0423 (15)	0.0554 (16)	−0.0025 (13)	−0.0069 (13)	−0.0034 (12)
C24	0.0361 (14)	0.0530 (17)	0.0594 (16)	−0.0039 (13)	−0.0010 (12)	−0.0085 (13)
C25	0.0361 (13)	0.0460 (15)	0.0621 (16)	−0.0125 (12)	0.0006 (12)	−0.0038 (13)
C26	0.0405 (13)	0.0481 (15)	0.0342 (12)	−0.0171 (12)	−0.0041 (10)	0.0013 (11)
C27	0.0553 (16)	0.0537 (17)	0.0463 (14)	−0.0250 (14)	−0.0084 (12)	−0.0002 (12)
C28	0.0534 (17)	0.080 (2)	0.0530 (16)	−0.0391 (17)	−0.0104 (13)	0.0025 (15)
C29	0.0388 (14)	0.080 (2)	0.0559 (16)	−0.0245 (15)	−0.0060 (12)	0.0024 (15)
C30	0.0358 (14)	0.0642 (18)	0.0539 (16)	−0.0157 (13)	−0.0006 (12)	−0.0021 (13)
N8	0.0599 (14)	0.0644 (16)	0.0454 (13)	−0.0334 (13)	−0.0035 (11)	0.0019 (11)
N9	0.142 (3)	0.0666 (18)	0.0517 (15)	−0.0527 (19)	−0.0295 (16)	0.0160 (13)
N10	0.092 (2)	0.0799 (19)	0.0594 (15)	−0.0493 (17)	−0.0246 (14)	0.0083 (13)
C34	0.0394 (13)	0.0392 (13)	0.0367 (12)	−0.0101 (11)	−0.0057 (10)	−0.0006 (10)
N7A	0.038 (3)	0.055 (5)	0.069 (3)	−0.011 (3)	−0.007 (3)	−0.014 (3)
C31A	0.033 (3)	0.035 (3)	0.036 (2)	−0.012 (2)	−0.0042 (18)	−0.0033 (17)
C32A	0.039 (4)	0.050 (4)	0.030 (3)	−0.013 (3)	−0.008 (2)	−0.005 (2)
C33A	0.045 (4)	0.042 (3)	0.032 (4)	−0.012 (4)	−0.009 (3)	0.001 (2)
C35A	0.046 (3)	0.047 (4)	0.0359 (16)	−0.020 (3)	−0.0114 (18)	−0.001 (2)
C36A	0.041 (3)	0.049 (4)	0.029 (3)	−0.017 (3)	−0.006 (2)	0.001 (2)
C37A	0.035 (2)	0.043 (3)	0.0364 (19)	−0.016 (2)	−0.0047 (16)	−0.0032 (17)
C38A	0.0355 (13)	0.0482 (15)	0.0483 (14)	−0.0143 (12)	−0.0043 (11)	−0.0031 (12)
C39A	0.031 (3)	0.030 (3)	0.0401 (17)	−0.011 (3)	0.0026 (17)	−0.0086 (18)
N7B	0.038 (3)	0.055 (5)	0.069 (3)	−0.011 (3)	−0.007 (3)	−0.014 (3)
C31B	0.033 (3)	0.035 (3)	0.036 (2)	−0.012 (2)	−0.0042 (18)	−0.0033 (17)
C32B	0.039 (4)	0.050 (4)	0.030 (3)	−0.013 (3)	−0.008 (2)	−0.005 (2)
C33B	0.045 (4)	0.042 (3)	0.032 (4)	−0.012 (4)	−0.009 (3)	0.001 (2)
C35B	0.046 (3)	0.047 (4)	0.0359 (16)	−0.020 (3)	−0.0114 (18)	−0.001 (2)
C36B	0.041 (3)	0.049 (4)	0.029 (3)	−0.017 (3)	−0.006 (2)	0.001 (2)
C37B	0.035 (2)	0.043 (3)	0.0364 (19)	−0.016 (2)	−0.0047 (16)	−0.0032 (17)
C38B	0.0355 (13)	0.0482 (15)	0.0483 (14)	−0.0143 (12)	−0.0043 (11)	−0.0031 (12)
C39B	0.031 (3)	0.030 (3)	0.0401 (17)	−0.011 (3)	0.0026 (17)	−0.0086 (18)
C40	0.0560 (15)	0.0388 (14)	0.0390 (13)	−0.0161 (12)	−0.0122 (11)	0.0017 (11)
C41	0.079 (2)	0.0384 (15)	0.0488 (16)	−0.0259 (14)	−0.0176 (14)	0.0023 (12)
C42	0.0624 (17)	0.0473 (15)	0.0413 (13)	−0.0254 (14)	−0.0096 (12)	0.0059 (12)
N11	0.0596 (16)	0.0695 (18)	0.0756 (18)	−0.0043 (13)	−0.0215 (14)	−0.0143 (14)
N12	0.0734 (18)	0.0589 (17)	0.0824 (19)	0.0006 (14)	−0.0258 (15)	0.0092 (14)
C43	0.0384 (13)	0.0483 (15)	0.0435 (13)	−0.0131 (12)	−0.0070 (11)	0.0088 (11)
C44	0.0456 (14)	0.0496 (16)	0.0467 (14)	−0.0094 (13)	−0.0167 (12)	0.0129 (12)
C45	0.0442 (14)	0.0572 (17)	0.0409 (13)	−0.0121 (13)	−0.0133 (11)	0.0056 (12)
C46	0.0456 (14)	0.0464 (16)	0.0523 (15)	−0.0122 (13)	−0.0130 (12)	0.0081 (12)
C47	0.0385 (14)	0.0469 (16)	0.0645 (17)	−0.0058 (12)	−0.0121 (13)	0.0001 (13)
N13	0.0707 (17)	0.0765 (19)	0.0655 (16)	−0.0187 (15)	−0.0198 (14)	0.0149 (14)
N14	0.143 (3)	0.0602 (18)	0.0742 (19)	−0.0231 (18)	−0.0528 (19)	0.0038 (15)
C48	0.0482 (16)	0.0474 (16)	0.0648 (17)	−0.0135 (14)	−0.0119 (14)	0.0019 (14)
C49	0.0516 (15)	0.0598 (18)	0.0451 (14)	−0.0300 (14)	−0.0131 (12)	0.0167 (13)
C50	0.0590 (17)	0.0601 (18)	0.0457 (15)	−0.0269 (15)	−0.0214 (13)	0.0201 (13)
C51	0.0613 (17)	0.0618 (19)	0.0408 (14)	−0.0287 (15)	−0.0180 (13)	0.0142 (13)
C52	0.0638 (18)	0.0572 (18)	0.0501 (15)	−0.0298 (15)	−0.0199 (13)	0.0161 (13)
C53	0.0595 (17)	0.0591 (18)	0.0515 (16)	−0.0238 (15)	−0.0156 (14)	0.0126 (14)
C54	0.090 (2)	0.0494 (17)	0.0608 (18)	−0.0253 (17)	−0.0302 (17)	0.0153 (14)

### Geometric parameters (Å, º)

**Table:**

O1A—H1A	0.844 (10)	C24—C25	1.382 (4)
O1A—H1B	0.837 (10)	C24—H24	0.9500
O1B—H1C	0.844 (10)	C25—H25	0.9500
O1B—H1D	0.845 (10)	C26—C27	1.385 (3)
O2A—H2A	0.852 (10)	C27—C28	1.394 (4)
O2A—H2B	0.850 (10)	C27—H27	0.9500
O2B—H2C	0.8508	C28—C29	1.362 (4)
O2B—H2D	0.8509	C28—H28	0.9500
O3A—H3A	0.853 (10)	C29—C30	1.381 (4)
O3A—H3B	0.853 (10)	C29—H29	0.9500
O3B—H3C	0.853 (10)	C30—H30	0.9500
O3B—H3D	0.853 (10)	N8—C39B	1.121 (15)
Ni1—N6	2.078 (2)	N8—C39A	1.142 (5)
Ni1—N5	2.084 (2)	N9—C41	1.155 (3)
Ni1—N1	2.0897 (19)	N10—C42	1.147 (3)
Ni1—N3	2.0945 (19)	C34—C35B	1.409 (16)
Ni1—N4	2.103 (2)	C34—C35A	1.410 (6)
Ni1—N2	2.1093 (19)	C34—C33A	1.412 (6)
N1—C5	1.336 (3)	C34—C33B	1.416 (16)
N1—C1	1.346 (3)	C34—C40	1.440 (3)
N2—C6	1.340 (3)	N7A—C38A	1.137 (5)
N2—C10	1.343 (3)	C31A—C36A	1.405 (9)
N3—C11	1.339 (3)	C31A—C32A	1.422 (8)
N3—C15	1.348 (3)	C31A—C37A	1.527 (7)
N4—C20	1.334 (3)	C32A—C33A	1.39 (2)
N4—C16	1.344 (3)	C32A—H32A	0.9500
N5—C25	1.338 (3)	C33A—H33A	0.9500
N5—C21	1.350 (3)	C35A—C36A	1.375 (6)
N6—C30	1.338 (3)	C35A—H35A	0.9500
N6—C26	1.344 (3)	C36A—H36A	0.9500
C1—C2	1.385 (3)	C37A—C39A	1.477 (6)
C1—C6	1.484 (3)	C37A—C38A	1.485 (4)
C2—C3	1.388 (4)	C37A—C37A^i^	1.653 (11)
C2—H2	0.9500	N7B—C38B	1.123 (15)
C3—C4	1.367 (4)	C31B—C32B	1.33 (2)
C3—H3	0.9500	C31B—C37B	1.39 (3)
C4—C5	1.390 (4)	C31B—C36B	1.39 (3)
C4—H4	0.9500	C32B—C33B	1.32 (8)
C5—H5	0.9500	C32B—H32B	0.9500
C6—C7	1.391 (3)	C33B—H33B	0.9500
C7—C8	1.380 (4)	C35B—C36B	1.368 (16)
C7—H7	0.9500	C35B—H35B	0.9500
C8—C9	1.375 (4)	C36B—H36B	0.9500
C8—H8	0.9500	C37B—C39B	1.404 (19)
C9—C10	1.374 (4)	C37B—C38B	1.490 (11)
C9—H9	0.9500	C40—C41	1.398 (3)
C10—H10	0.9500	C40—C42	1.411 (3)
C11—C12	1.392 (3)	N11—C47	1.146 (3)
C11—C16	1.487 (3)	N12—C48	1.140 (3)
C12—C13	1.382 (4)	C43—C44	1.416 (3)
C12—H12	0.9500	C43—C46	1.418 (4)
C13—C14	1.372 (4)	C43—C45	1.421 (3)
C13—H13	0.9500	C44—C45^ii^	1.352 (4)
C14—C15	1.378 (4)	C44—H44	0.9500
C14—H14	0.9500	C45—C44^ii^	1.352 (4)
C15—H15	0.9500	C45—H45	0.9500
C16—C17	1.386 (4)	C46—C47	1.412 (4)
C17—C18	1.377 (4)	C46—C48	1.424 (4)
C17—H17	0.9500	N13—C53	1.151 (3)
C18—C19	1.360 (4)	N14—C54	1.143 (4)
C18—H18	0.9500	C49—C52	1.415 (4)
C19—C20	1.388 (4)	C49—C50	1.417 (4)
C19—H19	0.9500	C49—C51	1.421 (3)
C20—H20	0.9500	C50—C51^iii^	1.359 (4)
C21—C22	1.378 (3)	C50—H50	0.9500
C21—C26	1.488 (3)	C51—C50^iii^	1.359 (4)
C22—C23	1.382 (4)	C51—H51	0.9500
C22—H22	0.9500	C52—C53	1.412 (4)
C23—C24	1.371 (4)	C52—C54	1.422 (4)
C23—H23	0.9500		
			
H1A—O1A—H1B	106.7 (17)	C24—C23—H23	120.3
H1C—O1B—H1D	105.8 (17)	C22—C23—H23	120.3
H2A—O2A—H2B	104.4 (16)	C23—C24—C25	118.6 (2)
H2C—O2B—H2D	104.3	C23—C24—H24	120.7
H3A—O3A—H3B	103.5 (17)	C25—C24—H24	120.7
H3C—O3B—H3D	103.8 (17)	N5—C25—C24	123.0 (2)
N6—Ni1—N5	78.69 (8)	N5—C25—H25	118.5
N6—Ni1—N1	169.09 (7)	C24—C25—H25	118.5
N5—Ni1—N1	96.11 (7)	N6—C26—C27	121.8 (2)
N6—Ni1—N3	96.17 (8)	N6—C26—C21	115.2 (2)
N5—Ni1—N3	93.51 (8)	C27—C26—C21	123.1 (2)
N1—Ni1—N3	93.69 (7)	C26—C27—C28	118.3 (3)
N6—Ni1—N4	96.76 (8)	C26—C27—H27	120.8
N5—Ni1—N4	170.39 (7)	C28—C27—H27	120.8
N1—Ni1—N4	89.75 (8)	C29—C28—C27	119.7 (3)
N3—Ni1—N4	78.47 (7)	C29—C28—H28	120.1
N6—Ni1—N2	92.20 (8)	C27—C28—H28	120.1
N5—Ni1—N2	94.96 (8)	C28—C29—C30	118.8 (3)
N1—Ni1—N2	78.61 (7)	C28—C29—H29	120.6
N3—Ni1—N2	169.11 (8)	C30—C29—H29	120.6
N4—Ni1—N2	93.66 (8)	N6—C30—C29	122.4 (3)
C5—N1—C1	118.4 (2)	N6—C30—H30	118.8
C5—N1—Ni1	126.90 (17)	C29—C30—H30	118.8
C1—N1—Ni1	114.74 (14)	C35A—C34—C33A	116.5 (11)
C6—N2—C10	118.3 (2)	C35B—C34—C33B	116 (3)
C6—N2—Ni1	113.74 (14)	C35B—C34—C40	117.9 (15)
C10—N2—Ni1	127.01 (18)	C35A—C34—C40	123.0 (5)
C11—N3—C15	118.2 (2)	C33A—C34—C40	120.3 (9)
C11—N3—Ni1	114.60 (15)	C33B—C34—C40	124 (3)
C15—N3—Ni1	126.56 (17)	C36A—C31A—C32A	117.3 (7)
C20—N4—C16	118.7 (2)	C36A—C31A—C37A	120.8 (5)
C20—N4—Ni1	126.95 (18)	C32A—C31A—C37A	121.8 (6)
C16—N4—Ni1	114.24 (15)	C33A—C32A—C31A	121.3 (9)
C25—N5—C21	117.9 (2)	C33A—C32A—H32A	119.4
C25—N5—Ni1	126.20 (17)	C31A—C32A—H32A	119.4
C21—N5—Ni1	115.10 (15)	C32A—C33A—C34	121.1 (15)
C30—N6—C26	118.9 (2)	C32A—C33A—H33A	119.4
C30—N6—Ni1	125.19 (18)	C34—C33A—H33A	119.4
C26—N6—Ni1	114.65 (15)	C36A—C35A—C34	123.1 (9)
N1—C1—C2	121.8 (2)	C36A—C35A—H35A	118.5
N1—C1—C6	115.7 (2)	C34—C35A—H35A	118.5
C2—C1—C6	122.5 (2)	C35A—C36A—C31A	120.6 (9)
C1—C2—C3	119.0 (2)	C35A—C36A—H36A	119.7
C1—C2—H2	120.5	C31A—C36A—H36A	119.7
C3—C2—H2	120.5	C39A—C37A—C38A	107.7 (4)
C4—C3—C2	119.5 (3)	C39A—C37A—C31A	110.8 (4)
C4—C3—H3	120.3	C38A—C37A—C31A	111.4 (3)
C2—C3—H3	120.3	C39A—C37A—C37A^i^	105.6 (4)
C3—C4—C5	118.4 (2)	C38A—C37A—C37A^i^	107.4 (3)
C3—C4—H4	120.8	C31A—C37A—C37A^i^	113.6 (3)
C5—C4—H4	120.8	N7A—C38A—C37A	175.9 (4)
N1—C5—C4	123.0 (2)	N8—C39A—C37A	176.7 (8)
N1—C5—H5	118.5	C32B—C31B—C37B	120 (2)
C4—C5—H5	118.5	C32B—C31B—C36B	121 (3)
N2—C6—C7	121.7 (2)	C37B—C31B—C36B	119 (2)
N2—C6—C1	115.8 (2)	C33B—C32B—C31B	121 (3)
C7—C6—C1	122.4 (2)	C33B—C32B—H32B	119.6
C8—C7—C6	118.9 (3)	C31B—C32B—H32B	119.6
C8—C7—H7	120.6	C32B—C33B—C34	122 (5)
C6—C7—H7	120.6	C32B—C33B—H33B	119.0
C9—C8—C7	119.4 (3)	C34—C33B—H33B	119.0
C9—C8—H8	120.3	C36B—C35B—C34	119 (3)
C7—C8—H8	120.3	C36B—C35B—H35B	120.7
C10—C9—C8	118.5 (2)	C34—C35B—H35B	120.7
C10—C9—H9	120.8	C35B—C36B—C31B	120 (3)
C8—C9—H9	120.8	C35B—C36B—H36B	119.9
N2—C10—C9	123.1 (3)	C31B—C36B—H36B	119.9
N2—C10—H10	118.5	C31B—C37B—C39B	119.6 (16)
C9—C10—H10	118.5	C31B—C37B—C38B	125.5 (11)
N3—C11—C12	122.1 (2)	C39B—C37B—C38B	114.3 (15)
N3—C11—C16	115.6 (2)	N7B—C38B—C37B	174.0 (15)
C12—C11—C16	122.4 (2)	N8—C39B—C37B	175.2 (19)
C13—C12—C11	118.6 (3)	C41—C40—C42	116.2 (2)
C13—C12—H12	120.7	C41—C40—C34	122.5 (2)
C11—C12—H12	120.7	C42—C40—C34	121.2 (2)
C14—C13—C12	119.6 (3)	N9—C41—C40	178.1 (4)
C14—C13—H13	120.2	N10—C42—C40	179.3 (3)
C12—C13—H13	120.2	C44—C43—C46	122.2 (2)
C13—C14—C15	118.7 (3)	C44—C43—C45	117.0 (2)
C13—C14—H14	120.6	C46—C43—C45	120.8 (2)
C15—C14—H14	120.6	C45^ii^—C44—C43	121.7 (2)
N3—C15—C14	122.7 (3)	C45^ii^—C44—H44	119.2
N3—C15—H15	118.6	C43—C44—H44	119.2
C14—C15—H15	118.6	C44^ii^—C45—C43	121.3 (2)
N4—C16—C17	121.5 (2)	C44^ii^—C45—H45	119.3
N4—C16—C11	115.9 (2)	C43—C45—H45	119.3
C17—C16—C11	122.6 (2)	C47—C46—C43	121.2 (2)
C18—C17—C16	118.7 (3)	C47—C46—C48	116.7 (2)
C18—C17—H17	120.6	C43—C46—C48	122.0 (2)
C16—C17—H17	120.6	N11—C47—C46	178.8 (3)
C19—C18—C17	120.2 (3)	N12—C48—C46	178.9 (3)
C19—C18—H18	119.9	C52—C49—C50	121.7 (2)
C17—C18—H18	119.9	C52—C49—C51	121.2 (2)
C18—C19—C20	118.3 (3)	C50—C49—C51	117.1 (3)
C18—C19—H19	120.8	C51^iii^—C50—C49	121.4 (2)
C20—C19—H19	120.8	C51^iii^—C50—H50	119.3
N4—C20—C19	122.5 (3)	C49—C50—H50	119.3
N4—C20—H20	118.7	C50^iii^—C51—C49	121.6 (2)
C19—C20—H20	118.7	C50^iii^—C51—H51	119.2
N5—C21—C22	122.1 (2)	C49—C51—H51	119.2
N5—C21—C26	114.3 (2)	C53—C52—C49	122.6 (2)
C22—C21—C26	123.6 (2)	C53—C52—C54	116.3 (3)
C21—C22—C23	119.1 (2)	C49—C52—C54	121.1 (2)
C21—C22—H22	120.5	N13—C53—C52	179.8 (3)
C23—C22—H22	120.5	N14—C54—C52	179.2 (3)
C24—C23—C22	119.3 (3)		
			
C5—N1—C1—C2	−1.3 (4)	Ni1—N6—C26—C21	−14.8 (2)
Ni1—N1—C1—C2	178.1 (2)	N5—C21—C26—N6	16.4 (3)
C5—N1—C1—C6	179.8 (2)	C22—C21—C26—N6	−162.5 (2)
Ni1—N1—C1—C6	−0.8 (3)	N5—C21—C26—C27	−163.0 (2)
N1—C1—C2—C3	0.2 (4)	C22—C21—C26—C27	18.1 (4)
C6—C1—C2—C3	179.0 (2)	N6—C26—C27—C28	2.1 (4)
C1—C2—C3—C4	0.9 (4)	C21—C26—C27—C28	−178.5 (2)
C2—C3—C4—C5	−0.7 (4)	C26—C27—C28—C29	0.7 (4)
C1—N1—C5—C4	1.5 (4)	C27—C28—C29—C30	−2.4 (4)
Ni1—N1—C5—C4	−177.81 (19)	C26—N6—C30—C29	1.3 (4)
C3—C4—C5—N1	−0.4 (4)	Ni1—N6—C30—C29	−165.05 (19)
C10—N2—C6—C7	−3.5 (4)	C28—C29—C30—N6	1.5 (4)
Ni1—N2—C6—C7	166.0 (2)	C36A—C31A—C32A—C33A	−3.8 (8)
C10—N2—C6—C1	177.2 (2)	C37A—C31A—C32A—C33A	173.1 (7)
Ni1—N2—C6—C1	−13.3 (3)	C31A—C32A—C33A—C34	2.9 (13)
N1—C1—C6—N2	9.6 (3)	C35A—C34—C33A—C32A	−0.5 (14)
C2—C1—C6—N2	−169.3 (2)	C40—C34—C33A—C32A	−174.6 (7)
N1—C1—C6—C7	−169.7 (2)	C33A—C34—C35A—C36A	−1.0 (13)
C2—C1—C6—C7	11.4 (4)	C40—C34—C35A—C36A	173.0 (6)
N2—C6—C7—C8	0.9 (4)	C34—C35A—C36A—C31A	0.1 (11)
C1—C6—C7—C8	−179.8 (3)	C32A—C31A—C36A—C35A	2.3 (7)
C6—C7—C8—C9	2.4 (5)	C37A—C31A—C36A—C35A	−174.7 (6)
C7—C8—C9—C10	−3.0 (5)	C36A—C31A—C37A—C39A	−21.1 (6)
C6—N2—C10—C9	2.8 (4)	C32A—C31A—C37A—C39A	162.1 (5)
Ni1—N2—C10—C9	−165.1 (2)	C36A—C31A—C37A—C38A	−140.9 (4)
C8—C9—C10—N2	0.4 (5)	C32A—C31A—C37A—C38A	42.3 (5)
C15—N3—C11—C12	−3.0 (4)	C36A—C31A—C37A—C37A^i^	97.6 (5)
Ni1—N3—C11—C12	168.3 (2)	C32A—C31A—C37A—C37A^i^	−79.2 (5)
C15—N3—C11—C16	177.6 (2)	C37B—C31B—C32B—C33B	177 (3)
Ni1—N3—C11—C16	−11.1 (3)	C36B—C31B—C32B—C33B	−2 (3)
N3—C11—C12—C13	0.1 (4)	C31B—C32B—C33B—C34	−6 (5)
C16—C11—C12—C13	179.5 (3)	C35B—C34—C33B—C32B	14 (5)
C11—C12—C13—C14	2.8 (5)	C40—C34—C33B—C32B	179 (2)
C12—C13—C14—C15	−2.8 (5)	C33B—C34—C35B—C36B	−15 (5)
C11—N3—C15—C14	3.0 (4)	C40—C34—C35B—C36B	179 (2)
Ni1—N3—C15—C14	−167.2 (2)	C34—C35B—C36B—C31B	8 (4)
C13—C14—C15—N3	−0.1 (4)	C32B—C31B—C36B—C35B	1 (2)
C20—N4—C16—C17	0.1 (4)	C37B—C31B—C36B—C35B	−178 (2)
Ni1—N4—C16—C17	−177.2 (2)	C32B—C31B—C37B—C39B	179.1 (18)
C20—N4—C16—C11	−179.1 (2)	C36B—C31B—C37B—C39B	−2 (2)
Ni1—N4—C16—C11	3.6 (3)	C32B—C31B—C37B—C38B	8.8 (19)
N3—C11—C16—N4	5.0 (3)	C36B—C31B—C37B—C38B	−172.0 (14)
C12—C11—C16—N4	−174.4 (2)	C35B—C34—C40—C41	179 (2)
N3—C11—C16—C17	−174.2 (2)	C35A—C34—C40—C41	−169.8 (6)
C12—C11—C16—C17	6.4 (4)	C33A—C34—C40—C41	3.9 (8)
N4—C16—C17—C18	−1.3 (4)	C33B—C34—C40—C41	14 (2)
C11—C16—C17—C18	177.9 (2)	C35B—C34—C40—C42	−6 (2)
C16—C17—C18—C19	1.4 (5)	C35A—C34—C40—C42	5.4 (7)
C17—C18—C19—C20	−0.4 (5)	C33A—C34—C40—C42	179.2 (7)
C16—N4—C20—C19	1.0 (4)	C33B—C34—C40—C42	−171 (2)
Ni1—N4—C20—C19	177.8 (2)	C46—C43—C44—C45^ii^	178.4 (3)
C18—C19—C20—N4	−0.8 (4)	C45—C43—C44—C45^ii^	0.0 (4)
C25—N5—C21—C22	−1.5 (3)	C44—C43—C45—C44^ii^	0.0 (4)
Ni1—N5—C21—C22	169.07 (18)	C46—C43—C45—C44^ii^	−178.4 (3)
C25—N5—C21—C26	179.6 (2)	C44—C43—C46—C47	−179.7 (2)
Ni1—N5—C21—C26	−9.9 (2)	C45—C43—C46—C47	−1.4 (4)
N5—C21—C22—C23	1.0 (4)	C44—C43—C46—C48	−2.4 (4)
C26—C21—C22—C23	179.8 (2)	C45—C43—C46—C48	175.9 (2)
C21—C22—C23—C24	0.5 (4)	C52—C49—C50—C51^iii^	−179.5 (3)
C22—C23—C24—C25	−1.4 (4)	C51—C49—C50—C51^iii^	−0.6 (4)
C21—N5—C25—C24	0.5 (4)	C52—C49—C51—C50^iii^	179.5 (3)
Ni1—N5—C25—C24	−168.87 (19)	C50—C49—C51—C50^iii^	0.6 (4)
C23—C24—C25—N5	0.9 (4)	C50—C49—C52—C53	−1.1 (4)
C30—N6—C26—C27	−3.1 (3)	C51—C49—C52—C53	−179.9 (2)
Ni1—N6—C26—C27	164.63 (18)	C50—C49—C52—C54	178.3 (3)
C30—N6—C26—C21	177.5 (2)	C51—C49—C52—C54	−0.6 (4)

Symmetry codes: (i) −*x*+1, −*y*+1, −*z*+2; (ii) −*x*+1, −*y*+2, −*z*+1; (iii) −*x*+1, −*y*+1, −*z*+1.

### Hydrogen-bond geometry (Å, º)

**Table:**

*D*—H···*A*	*D*—H	H···*A*	*D*···*A*	*D*—H···*A*
O1*A*—H1*A*···N14^iii^	0.84 (1)	2.60 (2)	3.438 (8)	174 (8)
O1*A*—H1*B*···N10^iv^	0.84 (1)	2.14 (2)	2.933 (5)	159 (5)
O1*B*—H1*C*···N10^iv^	0.84 (1)	2.11 (2)	2.865 (5)	150 (4)
O2*A*—H2*A*···N9^v^	0.85 (1)	2.22 (1)	3.068 (4)	178 (4)
O2*A*—H2*B*···N13^vi^	0.85 (1)	2.15 (1)	2.993 (4)	173 (4)
O2*B*—H2*C*···N13^vi^	0.85	2.05	2.803 (16)	147
O3*A*—H3*A*···O2*A*	0.85 (1)	2.09 (2)	2.71 (2)	130 (3)
O3*A*—H3*B*···O1*A*	0.85 (1)	2.09 (2)	2.85 (2)	147 (5)
O3*A*—H3*B*···O1*B*	0.85 (1)	2.39 (4)	3.20 (2)	160 (6)
O3*B*—H3*C*···O1*B*	0.85 (1)	1.99 (2)	2.84 (2)	170 (9)
O3*B*—H3*D*···O2*A*	0.85 (1)	2.10 (2)	2.864 (16)	148 (5)
C4—H4···N11^vii^	0.95	2.58	3.350 (3)	138
C5—H5···N3	0.95	2.67	3.213 (3)	117
C7—H7···O1*B*^v^	0.95	2.53	3.418 (7)	156
C10—H10···N6	0.95	2.63	3.168 (3)	116
C12—H12···O2*B*^vii^	0.95	2.44	3.30 (2)	150
C15—H15···N5	0.95	2.65	3.188 (3)	117
C15—H15···N12^viii^	0.95	2.68	3.369 (4)	130
C20—H20···N2	0.95	2.69	3.227 (3)	116
C22—H22···O3*B*	0.95	2.48	3.366 (15)	155
C25—H25···N8^viii^	0.95	2.49	3.184 (3)	130
C27—H27···O3*A*	0.95	2.55	3.43 (2)	155
C27—H27···O3*B*	0.95	2.33	3.276 (18)	172
C29—H29···N8	0.95	2.67	3.432 (3)	137
C29—H29···N11^iii^	0.95	2.69	3.295 (4)	123
C30—H30···N11^iii^	0.95	2.63	3.279 (4)	126

Symmetry codes: (iii) −*x*+1, −*y*+1, −*z*+1; (iv) *x*, *y*+1, *z*; (v) −*x*, −*y*+1, −*z*+2; (vi) *x*−1, *y*+1, *z*; (vii) −*x*, −*y*+1, −*z*+1; (viii) *x*−1, *y*, *z*.
